# Application of PBL combined with traditional teaching in the Immunochemistry course

**DOI:** 10.1186/s12909-023-04678-3

**Published:** 2023-09-22

**Authors:** Pingping Song, Xiangchun Shen

**Affiliations:** 1 The High Educational Key Laboratory of Guizhou Province for Natural Medicinal Pharmacology and Druggability), The State Key Laboratory of Functions and Applications of Medicinal Plants (The High Efficacy Application of Natural Medicinal Resources Engineering Center of Guizhou Province, Guizhou Medical University, Guian New District, Guizhou, 550000 China; 2 Key Laboratory of Optimal Utilization of Natural Medicine Resources, The Union Key Laboratory of Guiyang City-Guizhou Medical University), School of Pharmaceutical Sciences, Guizhou Medical University, Guian New District, Guizhou, 550000 China; 3 The Biology and Engineering College, Guizhou Medical University, Guian New District, Guizhou, 550000 China

**Keywords:** PBL, Traditional expository model, Immunochemistry, Final exam, Undergraduate

## Abstract

**Background:**

The problem-based learning (PBL) model has been widely carried out in many fields of medical colleges and universities. Immunochemistry as a cross-disciplinary science plays a vital role in preventing the occurrence of diseases and bridging the development of Life Science and Medicine. But now the Immunochemistry course still lacks the teaching practice in PBL. To explore the significance of PBL applied in the Immunochemistry course, the effect of the PBL model on the learning of undergraduates majoring in Chemicobiology was systematically evaluated.

**Methods:**

The teaching objects were the undergraduates majoring in Chemicobiology from Guizhou Medical University. The PBL model was applied in the Immunochemistry course. 62 undergraduates in Grade 2018 were set as the control group and adopted the traditional expository model. 93 undergraduates in Grades 2019–2020 were separately set as the experimental groups, which adopted the PBL model based on traditional lecture-based learning. In the PBL model, six cases related to course contents were designed for the students to complete. The final exams of the undergraduates in Grades 2018–2020 were analyzed by the score ranges (< 60 points, 60–69 points, 70–79 points, and ≥ 80 points) and nonparametric test. Finally, the questionnaire survey about the teaching evaluation was performed in Grades 2019–2020.

**Results:**

In Grades 2019 and 2020, the excellent rates (≥ 80 points), pass rates (≥ 60 points), fail rates (< 60 points), and average scores of the undergraduates were separately about 29%, 91.11% and 93.75%, 6.25%, and 8.89%, and 72.55 and 74.45 points. But in Grade 2018, the excellent rate, pass rate, failure rate, and average score of the undergraduates were separately 9.68%, 59.68%, 40.32%, and 62.55 points. By the statistical analysis, it was found that the excellent rates (χ^2^ = 8.317, *P* < 0.005) and pass rates (χ^2^ = 24.52, *P* < 0.0001) in Grades 2018–2020 were different, of which Grade 2020were the highest (29.17%, 93.75%) and Grade 2018 was the lowest (9.68%, 59.68%). The average score, excellent rate, and pass rate in Grade 2018 had significant differences with Grade 2019 (*P* < 0.0001, *P* < 0.0167) and Grade 2020 (*P* < 0.001, *P* < 0.0167). The questionnaire survey also showed that the student’s learning interests, independent problem-solving ability, knowledge structure system, and scientific thought and teamwork awareness were enhanced. In Grades 2019 and 2020, the ICC (95% CI) of criterion validity and inter-rater reliability were separately 0.42/0.34 and 0.81/0.80 (*P* < 0.0001).

**Conclusion:**

The combination of PBL and traditional expository models played positive roles in the student’s learning in the Immunochemistry course.

## Background

In 1969, Dr. Barrows, as an American professor of Neurology at the Medical School of McMaster University, initiated the teaching model of problem-based learning (PBL). PBL is a new educational way based on the curriculum and learning feedback [1–3]. Under the correct guidance and promotion of teachers, the students will discuss a posed problem in groups, then find and solve the problem by collecting related materials together, thus cultivating the students’ ability to independent learning and the courage to innovate and explore. The PBL model emphasizes the active learning of students rather than traditional teacher-based teaching [4, 5]. It can design specific topics or case studies according to the characteristics of each discipline and engages the learners in complicated and meaningful problem situations. The learners can solve the problems through their independent inquiry and cooperation, to learn scientific knowledge behind the problems and form problem-solving skills and independent learning abilities. In clinical medicine, PBL is performed by the cases as the guide, problems as the basis, students as the main body, teacher-oriented heuristic education, and ability cultivation of students as the teaching goal [6, 7]. The essence is to play a guiding role in the learning and mobilize the initiative and enthusiasm of students, which is different from the simple case analysis. It is especially suitable for curriculum teaching that can combine theory with practice [8, 9]. Therefore, the PBL model has been widely promoted in the fields of basic medicine and clinical medicine in medical colleges and universities [10, 11].

The advantages of PBL are mainly three points as follows [12]: (1) It can create a relaxed and active learning environment, enabling the students to actively express their views, and meanwhile, it is easy to obtain information from other students and teachers. (2) It can expose more problems in the course on the spot, deepen the understanding of students for theoretical knowledge in the discussion, constantly discover and solve new problems, and shorten the learning process. (3) It is conducive to the cultivation of students’ comprehensive abilities, such as literature review, summary, comprehensive understanding, logical reasoning, oral expression, and lifelong learning ability. As an open teaching model, the successful implementation of the PBL model depends on two aspects. On the one hand, PBL has high requirements for teachers’ self-quality and teaching skills [13]. The teachers must acquire proficiency in the course content and related subject knowledge. They can flexibly use their knowledge to raise and solve problems, which needs the abilities of rigorous logical thinking and good organizational management. They also need to mobilize the students’ learning enthusiasm, make learning fun and control the class rhythm in the process. The basic premise of PBL teaching is that the teachers must be familiar with the teaching syllabus and students’ abilities, distinguish the main points and difficult points of the course, formulate a targeted discussion outline, and select appropriate learning cases. On the other hand, PBL requires the active cooperation of students [14]. The students need to commonly prepare the materials, search the literature with cases and discuss in class. These preparations need to take much more time and energy than ordinary classroom learning. Therefore, the students must be active learners, otherwise, it’s difficult to achieve the expected teaching effects and objectives. These requirements for the PBL model can not only promote the students to digest and organically integrate scattered knowledge points but also improve their abilities of self-directed learning and practice innovation [15]. But whether PBL can improve the student’s academic record is still a question that is being pondered all over the world. Edgar Dale as a famous American learning expert proposed the Cone of Learning, which found that the retention rate of knowledge in the mode of active learning was placed first [16]. The PBL is just such an active learning model which enables the students to actively seek knowledge and solutions around the problems. The educators from Washington University compared two PBL courses with traditional AP courses, to explore if PBL were effective in improving academic performance, especially working for students in different social classes [17, 18]. The results showed that in the AP exam, the passing rate of students who took the PBL courses was 8% higher than those who took traditional AP courses and increased to 10% after one year. For those schools and students with poorer educational resources, PBL is also one of the effective ways to improve academic performance [19]. These research results have reassured more teachers about PBL.

Immunochemistry, as a new cross-disciplinary science of Immunology, Biochemistry, Biophysics, and Medicine, plays important roles in preventing the occurrence and spread of diseases and bridging the development of Life Science and Medicine [20–22]. After a comprehensive search in the databases, such as Google Scholar, Science Direct, Wiley Online Library, PubMed and Springer, it was found that the Immunochemistry course still lacks the teaching practice in PBL. Hence, the PBL model is especially popularized in the Immunochemistry course. In 2019, the Immunochemistry course was officially opened in the Department of Chemical Biology, School of Biology and Engineering, Guizhou Medical University. Currently, the undergraduates of Chemicobiology in Grades 2018–2020 have completed the course learning and examination. For Grade 2018, the teachers mainly adopted the traditional expository model and common learning platforms (Super Star and Rain Classroom) to assist the learning of students. Due to abstruse immunological knowledge in the course, the students majoring in Chemicobiology felt that the learning contents were extremely boring and difficult to understand deeply. Some students produced emotional learning weariness, thus failing the final exam. To improve the students’ interest in this course, the PBL teaching model was initially established for the undergraduates of Grade 2019. According to curriculum characteristics and professional training objectives in medical colleges, the combination of PBL and traditional teaching model were performed, which tried to activate the learning motivation of students and promote them fully know the connection between theory and practice. Therefore, undergraduates majoring in Chemicobiology from different grades were used for the research objects. The effectiveness of PBL and traditional expository models on the learning of students was systematically evaluated by the final exam and questionnaire survey, and the significance of PBL applied in the Immunochemistry course would be explored.

## Experimental methods

### Participants

The experimental participants were undergraduates of Chemicobiology in Grades 2018, 2019, and 2020. The Immunochemistry course has applied the PBL model.

### Study design

The 62 undergraduates of Grade 2018 as the control group were given the lecture in the traditional expository model. The 45 undergraduates in Grade 2019 and 48 undergraduates in Grade 2020 were separately set as two experimental groups. These two groups were subjected to the united mode of PBL and traditional teaching. And, the two ways of final exam (qualitative research) and questionnaire survey (quasi-experimental research) were performed to evaluate the teaching and study effect, of which the undergraduates in Grade 2018 only took the final exam. In addition, the teachers in the department made collective lesson preparation and communicated with each other.

### Educational intervention

In the PBL model, the teachers need to design related cases according to the chapter’s contents. The students in the class were divided into 8–10 groups, of which 5–6 members were assigned to each group. The chairman and secretary in each group were recommended. The chairman would lead the members to analyze the cases and raise the questions layer-by-layer, and the secretary would make the records. Finally, the group members would commonly discuss and collect related materials, and make summarized reports and evaluations in class. Through an organic combination of self-directed learning and classroom teaching, the students need to digest theoretical knowledge in the course and complete the analysis of six cases (Table 1).

### Data collection

The score ranges of final exam in different grades were set as < 60 points, 60–69 points, 70–79 points, and ≥ 80 points. The questionnaire forms were drawn up with six items and distributed to the undergraduates in Grades 2019–2020. After the students finished the forms, the evaluation ratings and scores were classified into Strongly Agree (5), Agree (4), Partially Agree (3), Disagree (2), and Strongly Disagree (1).

### Statistical analysis

In this research, the variances about the final exam of every grade were not uniform, and the ranked data about the excellent rate and pass rate didn’t also conform to the normal distribution, so the non-parametric tests were carried out. The Kruskal-Wallis test was used to analyze the average scores in Grades 2018–2020, and the test level was α = 0.05, and *P* < 0.05 was statistically significant. The Chi-square test was used to analyze the excellent rate and pass rate in Grades 2018–2020. Three pairwise comparisons were performed, and the Bonferroni-corrected test level was α = 0.0167, and *P* < 0.00167 was statistically significant. The reliability of the questionnaire was analyzed by the ICC (95% CI) of Criterion validity and Inter-rater reliability.

**Table 1:** The case study in the Immunochemistry course based on the PBL model

Case NO.	Case Name
1	The development of the cowpox vaccine
2	IgA and IgG subclass deficiencies
3	The factor I Deficiency-uncontrolled complement activation leads to susceptibility to infection
4	Severe rheumatoid arthritis treated with anti-TNF-α antibodies
5	MHC class II deficiency
6	Innate resistance to HIV

## Results

### The analysis of the final exam

**Fig. 1:**
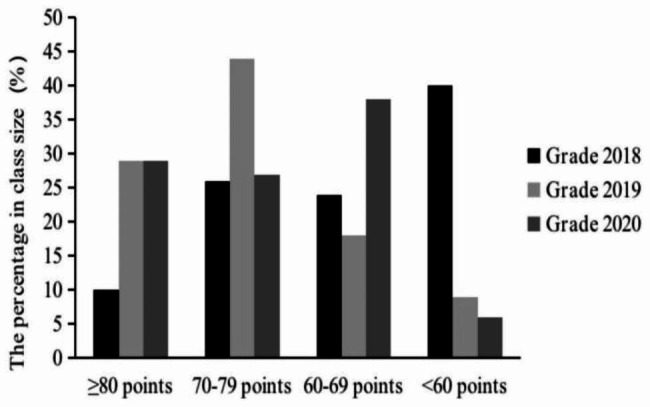
The proportional distribution of final exam scores in Grades 2018–2020

**Fig. 2:**
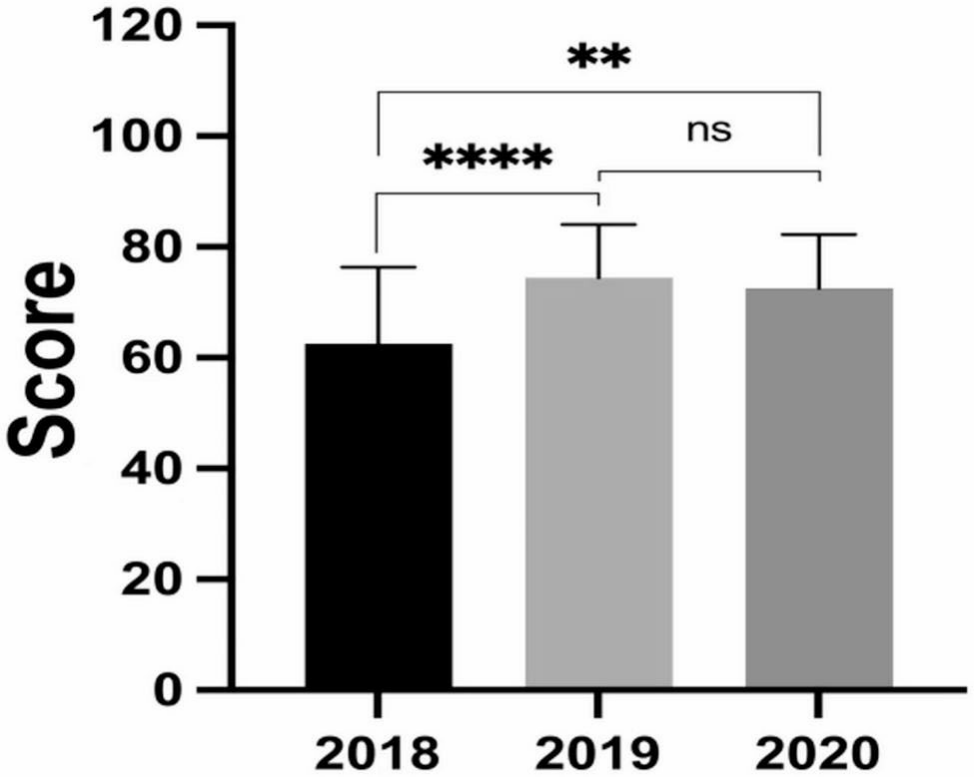
The average scores of the final exam in Grades 2018–2020. ****, **, and ns separately denotes *P* < 0.0001, *P* < 0.001, and *P* > 0.05

**Fig. 3:**
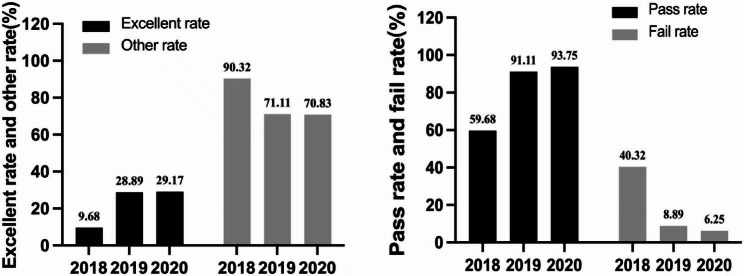
The excellent rates (≥ 80 points), pass rates (≥ 60 points) and fail rates (< 60 points) of the final exam in Grades 2018–2020

The final exam in Grades 2019–2020 were characterized in excellent rate (≥ 80 points), pass rate (≥ 60 points), fail rate (< 60 points), and average score. About 29% of the students in Grades 2019–2020 achieved excellent grades, and the pass rate, failure rate and average score were 91–94%, 6–9%, and 72.55–74.45 respectively. In Grade 2018, the excellent rate was 9.68%, and the pass rate, failure rate, and average score were 59.68%, 40.32%, and 62.55 respectively (Figs. 1, 2 and 3). From the score range of 60–79 points, 44% in Grade 2019 and 27% in Grade 2020 were in 70–79 points. 18% in Grade 2019 and 38% in Grade 2020 were in 60–69 points (Fig. 1).

In Grades 2018–2020, the excellent rates were different (χ^2^ = 8.317, *P* < 0.005), of which Grade 2020 was the highest (29.17%), then Grade 2019 was 28.89%, and Grade 2018 was the lowest (9.68%). The pass rates were different (χ^2^ = 24.52, *P* < 0.0001), of which Grade 2020 was the highest (93.75%), then Grade 2019 was 91.11%, and Grade 2018 was the lowest (59.68%). From the Kruskal-Wallis test, the average scores in Grade 2018 had a significant difference with Grade 2019 (Fig. 2, P < 0.0001) and Grade 2020 (Fig. 2, P < 0.001), while the difference between Grade 2019 and Grade 2020 was not significant (Fig. 2, P > 0.05). From the Chi-square test, the excellent rate and pass rate in Grade 2018 had significant differences with Grade 2019 (Fig. 3; Table 2, P < 0.0167) and Grade 2020 (Fig. 3; Table 2, P < 0.0167), while the difference between Grade 2019 and Grade 2020 was not significant (Fig. 3; Table 2, P > 0.0167).

### The analysis of the questionnaire survey

The questionnaire about the teaching evaluation showed the effect of the PBL model on the learning of undergraduates. Through the case analysis, classroom report, and self-evaluation, the student’s abilities in logical thinking, self-expression, and independent problem-solving were improved to a certain extent. Meanwhile, their cognition for knowledge learning and ability training, and the teamwork spirit among the members were also enhanced (Table 3). In Grades 2019 and 2020, the ICC (95% CI) of criterion validity and inter-rater reliability were separately 0.42/0.34 and 0.81/0.80 (*P* < 0.0001). Through the preparation of collective lessons and review of relevant literature, the interactive communication among the teachers was strengthened, the knowledge structure system was enriched and the teaching levels were also improved. In addition, the teachers could timely know the domestic and international research progress in the field, and obtain the latest knowledge, thus compensating for the slow updating of textbooks.

**Table 2:** The statistical analysis of excellent rate and pass rate in Grades 2018–2020

Grades	Excellent rate (%)	χ^2^	*P*	Pass rate (%)	χ^2^	*P*
2018	9.68	6.59	0.0103	59.68	13.04	0.0003
2019	28.89			91.11		
2018	9.68	6.908	0.0086	59.68	16.55	0.0001
2020	29.17			93.75		
2019	28.89	0.001	0.9765	91.11	0.23	0.6298
2020	29.17			93.75		

**Table 3:** The questionnaire survey about teaching evaluation in Grades 2019–2020

Items	N	Evaluation Ratings(N, %)				
		Strongly Agree	Agree	Partially Agree	Disagree	Strongly Disagree
Improved Logical Thinking	45 48	2(4.44%) 2(4.17%)	32(71.11%) 35(72.92%)	6(13.33%) 8(16.67%)	4(8.89%) 3(6.25%)	1(2.22%)
Improved Self-expression	45 48	4(8.89%) 3(6.25%)	26(57.78%) 29(60.42%)	10(22.22%) 9(18.75%)	5(11.11%) 7(14.58%)	
Improved Ability to Analyze and Solve the Problems Independently	45 48	5(11.11%) 7(14.58%)	36(80%) 38(79.17%)	4(8.89%) 3(6.25%)		
Enhanced Cognition for the Learning and Abilities Cultivation	45 48	1(2.22%) 2(4.17%)	30(66.67%) 31(64.58%)	10(22.22%) 12(25%)	4(8.89%) 3(6.25%)	
Enriched Knowledge Structure System	45 48	3(6.67%) 4(8.33%)	35(77.78%) 36(75%)	5(11.11%) 6(12.5%)	2(4.44%) 2(4.17%)	
Enhanced Team-work Spirit	45 48	40(88.89%) 42(87.5%)	3(6.67%) 4(8.33%)	2(4.44%) 2(4.17%)		
**Reliability**	**Grade**	**ICC (95% CI)**	***P***			
Criterion validity	2019 2020	0.42 (0.20–0.60) 0.34 (0.17–0.52)	< 0.0001			
Inter-rater reliability	2019 2020	0.81 (0.60–0.90) 0.80 (0.56–0.86)	< 0.0001			

## Discussion

In the age of artificial intelligence, information explosion, information acquisition, team cooperation, and problem-solving need to rely on reality rather than the textbook. Therefore, the current education should focus on encouraging students to understand society and master the ability to explore and solve problems. In this research, the effectiveness and significance of PBL application on the learning of undergraduates majoring in Chemicobiology were systematically evaluated by the Immunochemistry final exam and questionnaire survey. This will further provide theoretical support for the application of the PBL model in multiple subjects.

Compared with the traditional expository model, the application of the PBL model significantly improved the final exam scores of undergraduates in Immunochemistry. The teaching effect was mainly reflected in the average score, excellent rate, pass rate, and failure rate. The average score, excellent rates, and pass rates of students in Grades 2019 and 2020 were significantly higher than those in Grade 2018, and the failure rates were significantly lower than the control group (Figs. 2 and 3; Table 2). Noticeably, the excellent rate and pass rate between Grade 2019 and 2020 have no significant difference (Fig. 3; Table 2), but in the range of 70–79 points the student proportion of Grade 2019 was higher than Grade 2020, and in the range of 60–69 points conversely lower (Fig. 1). This difference might be induced by the increase in the number and difficulty of exam questions in the test system. Because the exam contents to be covered were expanded, the number of questions used for online random extraction was increased from 200 to 470. Besides this, the effect of the PBL application in Grades 2019–2020 was very good and was highly praised by the teachers and students (Table 3). It was seen that the connection between theory and practice, and problem discovery and solution are closely reflected in the Immunochemistry course [23]. This teaching demand is similar to the mission of the PBL model, so the PBL model will be in good agreement with Immunochemistry course. However, PBL aims to cultivate the students’ comprehensive ability, rather than improve the test scores, so there are still some selection limitations for the assessment indicators in this course.

Immunochemistry as an interdisciplinary subject uses abstract theories and methods of Chemistry to study the problems of Life Science. It can provide a theoretical basis for the development of new drugs and the prevention and cure of diseases by interfering with the pathogenesis of human diseases [24]. Therefore, quickly digesting and updating the theories in the Immunochemistry course is particularly important for the students to keep up with the times. With the progress of science and technology, the traditional expository model is hard to meet the students’ learning demand for academic frontiers knowledge. But it’s also difficult for students to read English articles and translate them into Chinese. The advantages of the PBL model with classic cases analysis and classroom reports are reflected in connecting complicated and scattered theories and organically integrating related knowledge involved in different disciplines, which will help the students to master the scientific knowledge behind the problems and form a systematic knowledge framework [25, 26]. The case analysis and discussion in each chapter will gradually improve the student’s ability in English reading and enrich their subject knowledge and scientific thought. The questionnaire survey also confirmed that the combination of PBL and the traditional expository model can activate the students’ enthusiasm and curiosity, promote the digestion of theoretical knowledge, and improve the abilities of self-directed learning and problem-solving independently. Besides these, the case analysis and group report can also cultivate the students’ self-expression and cooperative awareness. Hence, for these fields of Basic Medicine and Clinical Medicine in medical colleges and universities, the application of the PBL model plays positive and meaningful roles in improving the students’ classroom efficiency, knowledge reserve, and comprehensive abilities.

At present, the PBL model has been promoted in medical colleges and universities [27, 28], but there are still some problems in practical teaching, which are mainly embodied in four aspects. Firstly, practical and effective application is still scarce. The main reason is that the training objectives, teaching syllabus, and curriculum setting of different disciplines have great differences, and the teachers’ acceptance and appreciation for a new teaching model are far from enough. At Guizhou Medical University, Immunochemistry course is only given to undergraduates majoring in Chemical Biology and didn’t involve the other majors. Therefore, this study lacked the evaluation of the teaching effect in different majors of medical universities. Given the gradual revision of training programs, Immunochemistry course would be gradually opened in related majors of our university, such as Biotechnology and Biomedical Engineering. In the future, the teaching effect of the PBL model in other majors will be comprehensively compared. Secondly, Chinese students have been receiving a “cramming” education for a long time [29]. They have formed certain dependence on the traditional education model and cannot actively discover and solve problems. Some students are only satisfied with getting good scores, so they feel that the PBL model wastes too much time. Therefore, the students should change their mindset and complete the role transformation from passive learner to learning master. Thirdly, many colleges and universities set fixed teacher-student ratios based on the traditional expository model. It’s difficult for the PBL model to be effectively performed in classes with many students. The students may not independently acquire the knowledge and solve the problems through case analysis and group discussion [30, 31]. So the teaching of small classes with the PBL model should be promoted. Fourthly, less class time and scarce teaching resources in the curriculum sets aren’t conducive for students to devote themselves to learning [32]. Therefore, practical teaching and reasonable allocation of teaching resources must be emphasized, and the PBL model needs to be actively adopted to guide the students to learn efficiently. In short, the effect of the PBL model may take at least two years to show, so extended follow-up research and in-depth exploration are particularly necessary.

In PBL, the knowledge is from self-directed learning, and the reasoning skills were catalyzed by the questions. The students can actively learn how to find the information, explore the solutions, and adapt to cope with endless problems in future careers. This is in line with the nature of education and is also why the PBL model is increasingly accepted. In world-famous universities, the extracurricular comprehensive abilities of students are especially emphasized. Those factors that can’t be reflected in standardized scores, such as innovation, independent thinking, solutions, leadership, wisdom, and values, often play a decisive role in the selection of students.

## Conclusion

The combination of PBL and the traditional teaching model has a positive impact on the students’ learning in Immunochemistry course. The effectiveness of the PBL model was mainly reflected in improving the final exam scores, activating the students’ learning interest, promoting their abilities to solve problems independently, enriching their knowledge structure system, and significantly exercising their scientific thought and teamwork awareness.

## Data Availability

The datasets used and/or analyzed during the current study are available from the corresponding author upon reasonable request.
